# MALDI-TOF MS Analysis of Proanthocyanidins in Two Lowland Tropical Forest Species of *Cecropia*: A First Look at Their Chemical Structures

**DOI:** 10.3390/molecules190914484

**Published:** 2014-09-12

**Authors:** Alex Van Huynh, John M. Bevington

**Affiliations:** Department of Biological Sciences, Moravian College, 1200 Main Street, Bethlehem, PA 18018-6650, USA; E-Mail: jmb@cs.moravian.edu

**Keywords:** proanthocyanidins, condensed tannins, *Cecropia sciadophylla*, *Cecropia polystachya*, MALDI-TOF mass spectrometry, anti-herbivore defense tradeoffs

## Abstract

The structural chemistry of proanthocyanidin molecules has been investigated in temperate zone plants, but few studies have been done with plants of the Amazonian lowland tropical wet forests where herbivore pressure is more extensive and diverse. Using MALDI-TOF mass spectrometry, we report unique properties of the proanthocyanidin structural chemistry in two neotropical *Cecropia* species, *C. polystachya*, a myrmecophyte with mutualistic ants, and *C. sciadophylla*, a non-myrmecophyte lacking mutualistic ants. Our preliminary data suggests the presence of reportedly uncommon propelargonidin subunits in a majority of proanthocyanidin oligomers. The presence of 3-*O*-gallate proanthocyanidin monomers was also detected in the mass spectra of both species. Unlike other studies that have examined species growing at higher latitudes, oligomers composed of procyanidin, propelargonidin, and their 3-*O*-gallates were present in both *Cecropia* species while the presence of oligomers containing prodelphinidin units were absent or at lower levels. These distinctive features may suggest that proanthocyanidins in some tropical plant species may be an untapped source of proanthocyanidin structural complexity that warrants further investigation. Several differences between spectra of the two *Cecropia* species could also point to the presence of anti-herbivore defense tradeoffs between chemical defense quality and biotic defense between the two species.

## 1. Introduction

**Figure 1:**
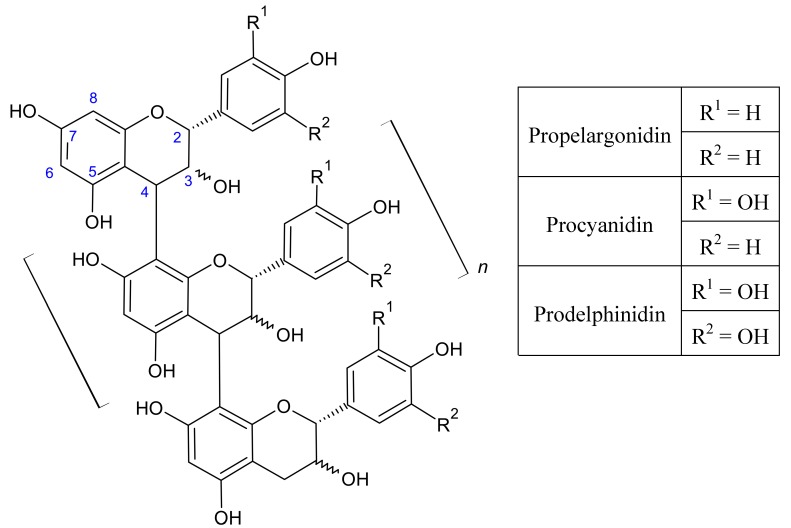
Proanthocyanidin polymer structure and monomeric flavan-3-ol precursors.

Proanthocyanidins, also known as condensed tannins, are natural polyphenolic plant products that seem to be produced in most vascular plants through a chloroplast-derived organelle recently described as the “tannosome” [1]. A principal role of these compounds is to act as deterrents to herbivory, probably functioning through protein binding via hydrogen and/or covalent bonding and through the production of oxygen radicals [2,3,4,5]. Proanthocyanidins are comprised of flavan-3-ol monomers, most notably catechin and epicatechin, commonly connected through C4-C8 bonds [6,7]. Proanthocyanidin structure has a large degree of potential variation such as hydroxyl substitution on the B ring, stereochemistry of the C ring, galloylation or glycosylation of the C ring, monomeric bonding patterns, and degree of polymerization (Figure 1). The unique composition of a given proanthocyanidin structure has been implicated in influencing its biological activity [8,9].

While proanthocyanidin structures and their effects on herbivores have been investigated primarily in temperate zone plants [8], with *Quercus* spp. being a notable example [10,11,12,13], proanthocyanidins in lowland wet forests of the Amazon where tree species diversity and herbivory are hyperdiverse have not been studied extensively [14].

Here we report the first investigation of proanthocyanidin structural chemistry in two lowland tropical species of *Cecropia*, a neotropical genus of 61 recognized tree species that occupy disturbed areas along riverbanks or in interior tree-fall gaps [15]. Most species are myrmecophytes that develop mutualistic relationships with ants, most notably *Azteca* species. The ants establish colonies in hollow internodes of the trees and defend the trees against herbivores. The trees in turn provide food for the ants in the form of glycogen-rich Müllerian bodies [15]. Some *Cecropia* species, however, are non-myrmecophytes and do not have ants [15]. In this study, we examined proanthocyanidin structures in *C. polystachya*, a myrmecophyte, and C*. sciadophylla*, a non-myrmecophyte.

Because proanthocyanidins may be an important component of chemical defense against herbivores in longer lived trees [16], we hypothesized that any differences in herbivore defense in these two species of *Cecropia* trees may manifest themselves not only in the presence or absence of mutualistic ants, but also in the structure of their proanthocyanidins. For this reason we used matrix-assisted laser desorption/ionization time of flight mass spectrometry (MALDI-TOF MS) and two dimensional thin layer chromatography (TLC) to examine the structural chemistry of proanthocyanidins of both mature and immature leaves of *C. sciadophylla* and *C. polystachya*.

## 2. Results and Discussion

**Figure 2:**
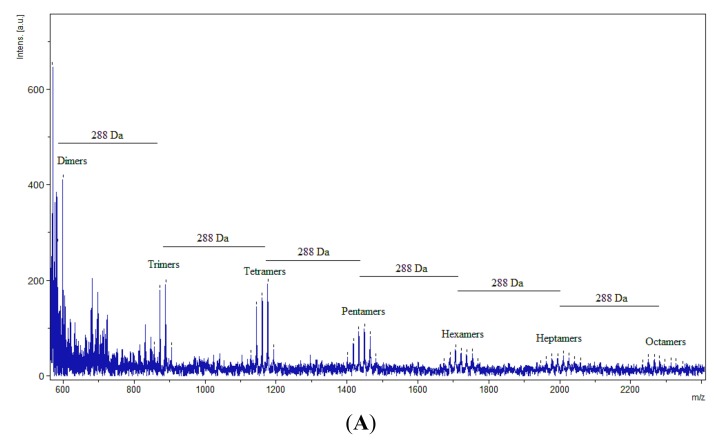

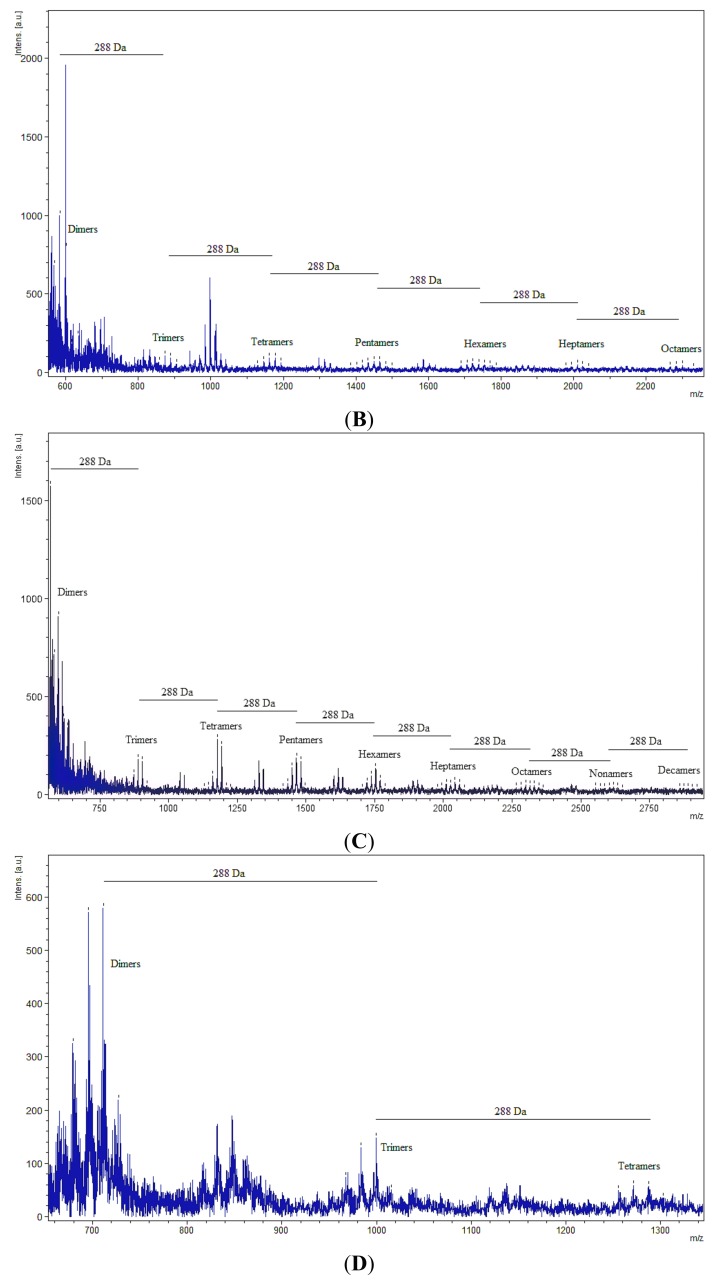
MALDI spectrum of proanthocyanidins extracted from *Cecropia* leaves. The distance between subsequent groups of peaks is 288 Da, the mass of an additional procyanidin unit. (**A**) *Cecropia sciadophylla* immature leaves (Na^+^). (**B**) *Cecropia sciadophylla* mature leaves (Na^+^). (**C**) *Cecropia polystachya* immature leaves (Na^+^). (**D**) *Cecropia polystachya* mature leaves (Cs^+^).

**Figure 3:**
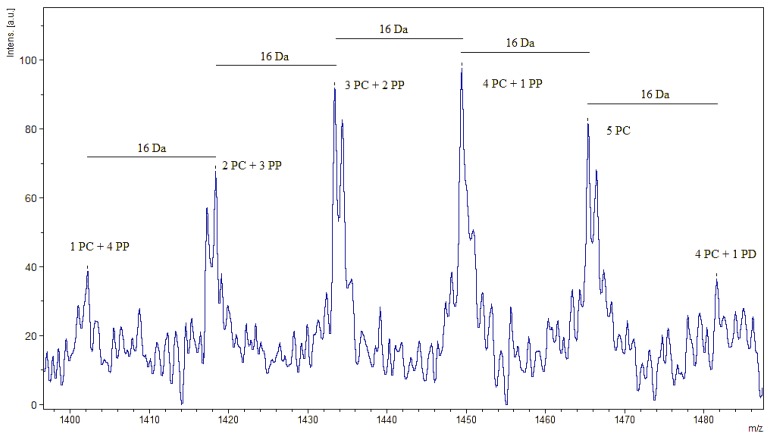
Zoomed image illustrating degree of substitution distribution patterns of *Cecropia sciadophylla* proanthocyanidins extracted from immature leaves. The distance between subsequent peaks is 16 Da, the mass of an additional hydroxyl group. PC: procyanidin, PD: prodelphinidin, PP: propelargonidin.

Resolution of proanthocyanidins by MALDI-TOF MS was comparable to the quality of spectra produced in other studies [17,18,19], supporting the reliability of the extraction procedure in its degree of purification. The characteristic feature marking the presence of proanthocyanidins is an arithmetic series of peaks, each peak 288 Da, the mass of an additional procyanidin unit (Figure 1), greater than the previous peak. Thus, each group of peaks represents an oligomer of one greater degree of polymerization than that of the previous peak 288 Da lighter. Proanthocyanidins from immature and mature leaves of *C. sciadophylla* were resolved up to an octamer (Figure 2A,B). Immature leaves of *C. polystachya* were resolved up to a decamer (Figure 2C). In contrast, the mature leaves of *C. polystachya* were only resolved up to a tetramer (Figure 2D). The peak of each oligomer of a specific degree of polymerization is further divided into a series of smaller sub-peaks (Figure 3).

This phenomenon can offer insights into the composition of the types of oligomers present. This is based on the fact that the different potential monomers (procyanidins, prodelphinidins, and propelargonidins) all differ by the number of hydroxyl groups they possess on the B ring. Thus, after calculating the theoretical mass number for a certain sized oligomer consisting entirely of procyanidins, the sub-peaks one finds that are multiples of 16 Da apart (the mass of an added/removed hydroxyl) correspond to the replacement of a procyanidin unit with either a prodelphinidin unit (the addition of a hydroxyl) or with a propelargonidin unit (the removal of a hydroxyl).

From the series of split sub-peaks 16 Da apart that are present for a certain oligomer, the monomeric composition can be inferred using the equation 288*a* + 304*b* + 272*c* + 2 + *d* where *a* is the number of procyanidin units, *b* is the number of prodelphinidin units, *c* is the number of propelargonidin units, 2 corresponds to the number of extra hydrogens on the terminal subunits, and *d* is the atomic mass of the ionizing agent (e.g., Na^+^). Using this analysis, the monomeric composition of the oligomer representative of each sub-peak can be calculated in the form of the number of each type of monomeric component (Table 1). Table 1 is representative of the remaining three samples.

Sub-splitting analysis revealed that the majority of peaks for all four samples represent oligomers contain procyanidin and propelargonidin units. Peaks representing the presence of prodelphinidin units for given oligomers in each of the four samples were in the minority if not absent altogether. The possibility of potassium as the ionizing agent is a potential confounding factor and this fact may account for the observation of prodelphinidin containing molecules since the replacement of Na^+^ with K^+^ as the ionizing agent would result in a 16 Da mass increase.

**Table 1:** Observed and calculated masses for *Cecropia sciadophylla* proanthocyanidins extracted from immature leaves. Mass calculations were based on the equation 288*a* + 304*b* + 272*c* + 2 + 23, where *a* is the number of procyanidin units, *b* is the number of prodelphinidin units, and *c* is the number of propelargonidin units, 2 corresponds to the number of extra hydrogens on the terminal subunits and 23 is the atomic mass of sodium.

Degrees of Polymerization	Number of Procyanidin Units (*a*)	Number of Prodelphinidin Units (*b*)	Number of Propelargonidin Units (*c*)	Calculated [M + Na]^+^	Observed [M + Na]^+^
Dimer	0	0	2	569	569
	1	0	1	585	585
	2	0	0	601	601
Trimer	1	0	2	857	857
	2	0	1	873	873
	3	0	0	889	889
	2	1	0	905	905
Tetramer	1	0	3	1129	1129
	2	0	2	1145	1145
	3	0	1	1161	1161
	4	0	0	1177	1177
	3	1	0	1193	1193
Pentamer	1	0	4	1401	1402
	2	0	3	1417	1418
	3	0	2	1433	1433
	4	0	1	1449	1449
	5	0	0	1465	1465
	4	1	0	1481	1482
Hexamer	1	0	5	1673	1674
	2	0	4	1689	1691
	3	0	3	1705	1706
	4	0	2	1721	1722
	5	0	1	1737	1738
	6	0	0	1753	1754
	5	1	0	1769	1770
Heptamer	1	0	6	1945	1947
	2	0	5	1961	1962
	3	0	4	1977	1978
	4	0	3	1993	1994
	5	0	2	2009	2010
	6	0	1	2025	2026
	7	0	0	2041	2042
	6	1	0	2057	2059
Octamer	2	0	6	2233	2233
	3	0	5	2249	2250
	4	0	4	2265	2267
	5	0	3	2281	2281
	6	0	2	2297	2296
	7	0	1	2313	2313
	8	0	0	2329	2329
	7	1	0	2345	2347

These findings were surprising because propelargonidin monomers are rare in nature and are not often seen in plant proanthocyanidins [6,7]. Ayres *et al.* [8] found no presence of propelargonidin units in 16 woody plant species over six families and Zhang and Lin [19], using MALDI-TOF MS, found that proanthocyanidins in Japanese Oak (*Lithocarpus glaber*) contained only procyanidin and prodelphinidin units. Behrens *et al.* [17], using MALDI-TOF MS, found that lime (*Tilia cordata*) and willow (*Salix alba*) appeared to contain only procyanidin units while spruce (*Picea abies*) contained mixtures of procyanidin and prodelphinidin units. Behrens et al [17] did find that beech (*Fagus sylvatica*) appeared to contain minor peaks reflecting the presence of propelargonidin units but these were not as prevalent as those reflecting a combination of procyanidin and prodelphinidin units. Although our data is strongly consistent with the presence of appreciable propelargonidin containing oligomers, without the benefit of HPLC, we propose a tentative assignment of propelargonidin containing proanthocyanidins in these two species of *Cecropia*.

**Figure 4:**
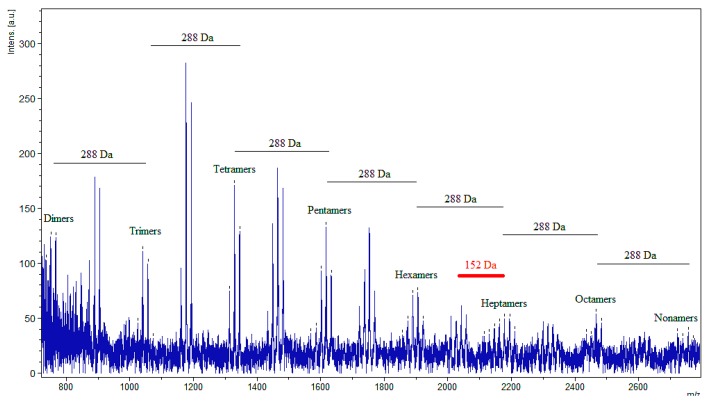
MALDI spectrum of *Cecropia polystachya* proanthocyanidins extracted from immature leaves. Displayed is the polymerization pattern for sodium ions with polymers possessing one galloyl ester (marked with ticks). The distance between subsequent groups of peaks is 288 Da, the mass of an additional procyanidin unit. The shifted distance from the original peak series is 152 Da, the mass of one added galloyl ester substituent.

Spectra for all samples also revealed the presence of oligomers containing at least one 3-*O*-gallate flavan-3-ol derivative (Figure 4). These subunits containing a galloyl ester off of carbon 3 on the C ring in place of a normal hydroxyl are visually discernible in the spectra by the presence of an arithmetic series of peaks shifted 152 Da upwards from the original non-3-*O*-gallate oligomeric series. While the presence of multiple subunits possessing a galloyl ester may be present in a single proanthocyanidin oligomer, the ability to discern the exact number is limited by the MALDI mass spectra. While the presence of one galloyl ester is easily seen via the peak series 152 Da greater than the original non-galloyl ester series, the presence of two galloyl esters would result in a shifted spectrum 304 Da upwards. This potential peak overlaps directly with a variant of the next original non-3-*O*-gallate containing oligomer. Thus, the presence of two galloyl esters on one oligomer and the next oligomer possessing one greater degree of polymerization mask each other. The peaks designated as representing an oligomer containing a prodelphinidin unit (Figure 3) may be a result of potassium ionization of a non-prodelphinidin containing oligomer or an oligomer containing two galloyl ester substituents.

**Figure 5:**
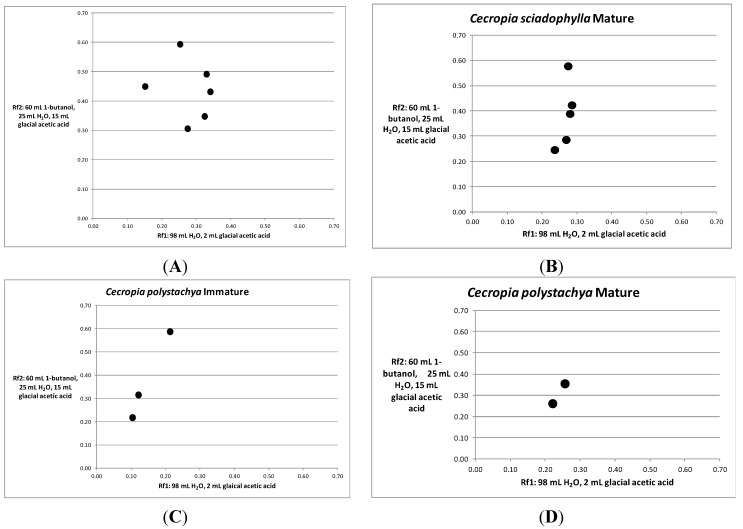
Two dimensional TLC of thiolyzed proanthocyanidins extracted from *Cecropia* leaves. (**A**) *Cecropia sciadophylla* immature leaves. (**B**) *Cecropia sciadophylla* mature leaves. (**C**) *Cecropia polystachya* immature leaves. (**D**) *Cecropia polystachya* mature leaves.

Thiolysis of each of the four samples yielded marked differences between species in the number of vanillin/HCl reaction spots. The immature *C. sciadophylla* leaf yielded six spots (Figure 5A) and the mature leaf yielded five spots (Figure 5B). In contrast, the immature *C. polystachya* leaf yielded three spots (Figure 5C) and the mature leaf yielded only two spots (Figure 5D). The heterogeneous proanthocyanidin composition in *C. sciadophylla* contrasts with temperate zone plants such as blackbrush (*Coleogyne ramosissima*) and low-brush cranberry (*Vacciniumvitis-idaea*), both of which possess proanthocyanidins comprised entirely of procyanidin units [8].

In comparing our results between the two species, TLC analysis of thiolyzed proanthocyanidins showed that the composition of proanthocyanidin monomers from both the immature and mature leaves of *C. sciadophylla* was more heterogeneous than from the leaves of *C. polystachya*. This is interesting because it might have ecological significance. *C. polystachya* is defended from herbivores by colonies of aggressive ants. Anti-herbivore defense in *C. sciadophylla* is related to the chemical and physical properties of its leaves. The greater chemical heterogeneity of the proanthocyanidin monomers in *C. sciadophylla* raises the possibility that the chemical properties of its proanthocyanidins may contribute to its anti-herbivore defense. We have previously found that the leaves of *C. sciadophylla*, particularly the immature leaves, contain high levels of chemical defense in the form of proanthocyanidins and protein precipitable phenolics, and that these levels are significantly greater than those found in *C. membranacea*, another myrmecophyte species [20]. These findings may reflect a tradeoff between biotic defense and chemical defense, especially in the immature leaves [21]. Such tradeoffs have been demonstrated with other genera containing ant mutualisms such as *Macaranga* [22], *Inga* [23], and *Acacia* [24]. Further work will be required to determine if this is the case.

## 3. Experimental

### 3.1. Sample Collection

Two *Cecropia* species, *C. sciadophylla* and *C. polystachya*, were collected in premontane forest along the Alto Madre de Dios River in Manu Park, Peru (12°39'170"S, 71°14'246"W; elevation of 437 m asl). Trees were approximately 1 km apart from each other and were chosen based on the presence of both mature and immature leaves. Leaves were harvested, measured, and dried in a plant press. Blotters and driers were changed frequently. Dry leaves were transferred in newspapers to large, air-tight Ziploc^®^ bags with silica gel contained in paper pouches.

### 3.2. Proanthocyanidin Extraction

Proanthocyanidin extraction procedures were adapted from extraction steps found in several studies examining plant proanthocyanidin structure [16,18,25,26,27]. Tissue samples (1 gm) of dry leaf tissue were cut into small strips, soaked overnight at 4 °C in 70% aqueous acetone, ground in chilled mortars, and extracted three times with cold 70% aqueous acetone (25 mL). The combined acetone extracts were subjected to rotary evaporation under reduced pressure at 35–40 °C to remove the acetone. Aqueous samples were then extracted twice in a separatory funnel with equal volumes of hexanes to remove lipophilic substances, ethyl acetate to remove chlorophyll, and chloroform to remove carotenoids and monomeric phenols. Each aqueous sample was again subjected to rotary evaporation under the same conditions to remove any residual organic solvent. The samples were then filtered through Number 43 Whatman filter paper. The samples were run through a Sephadex LH-20 liquid chromatography column. Carbohydrates and hydrolyzable tannins were eluted with 80% aqueous ethanol while proanthocyanidins adhered to the column. Proanthocyanidins were later eluted using 70% acetone. A final rotary evaporation resulted in aqueous proanthocyanidin extracts which were stored at 4 °C in plastic screw cap vials.

### 3.3. MADLI-TOF Mass Spectrometry

From reports of success with obtaining MALDI-TOF mass spectra of proanthocyanidins isolated from plant material, 2,5-dihydroxybenzoic acid (DHB, Aldrich, St. Louis, MO, USA) was selected as a matrix to crystallize the proanthocyanidin samples [17,18,19]. Ten mg of DHB was dissolved in 1 mL of deionized water and was mixed with the aqueous proanthocyanidin extracts from each species at a 3:1 volumetric ratio. A volume of 2 μL of each analyte-matrix solution was applied to a stainless steel target and allowed to dry and crystalize for 10–15 minutes before being subjected to irradiation and ionization with either sodium or cesium ions [28]. Samples were pulsed for 130 ns with a nitrogen laser (λ = 337nm) on a Bruker Daltonics Autoflex instrument (Bruker, Billerica, MA, USA). Spectra were taken in positive deflection mode with an accelerating voltage of 19.0 kV and a reflector voltage of 20.0 kV. Spectra were obtained from 100–150 shots and calibrated using ACTH 18–39 (2465.20 Da), angiotensin II (1046.54 Da), bradykinin 1–7 (757.40 Da), and P14R (1533.86 Da) (Bruker).

### 3.4. Thiolysis and Two Dimensional TLC

A volume of 200 μL of aqueous proanthocyanidin extracts was mixed with 200 μL of cysteamine thiolysis mixture (20 μL of concentrated HCl, 930 μL of methanol, and 50 mg of cysteamine hydrochloride) as described by Torres and Lozano [29]. Thiolysis was carried out in an 80 °C water bath in sealed water-tight microfuge tubes. The reaction was stopped with 1.2 mL of 0.1% aqueous trifluoroacetic acid after 4 hours. A 25 μL sample of the thiolysis product, applied in 2 μL applications, was analyzed via TLC on 20 × 20 cm cellulose plates (Merck KGaA, Darmstadt, Germany). The first dimension solvent was deionized water (98 mL) and glacial acetic acid (2 mL). The second dimension solvent was 1-butanol (60 mL), deionized water (25 mL), and glacial acetic acid (15 mL) [30]. Completed plates were sprayed with a vanillin/HCl reagent (1 gm vanillin in 10 mL of concentrated HCl). This spray specifically reacts with proanthocyanidins and their monomeric components to produce red/pink spots [6,29].

## 4. Conclusions

Here we report a first look at proanthocyanidin structure in *Cecropia* and one of the few investigations of proanthocyanidins in tropical plants of the Amazonian lowland wet forests. Our data suggest a unique feature of multiple propelargonidin units in *Cecropia* proanthocyanidins, the presence of galloylated monomers, and a relatively high level of heterogeneity in proanthocyanidin composition compared to temperate zone plants previously reported. Differences in proanthocyanidin composition between the myrmecophyte and non-myrmecophyte *Cecropia* species were also observed, specifically in number of monomeric components. These differences are intriguing, as they may reflect tradeoffs in anti-herbivore defense that are a function of the level of biotic defense. Further chemical analysis such as MALDI-TOF MS post source decay analysis, HPLC, and NMR studies can confirm the presence of the suggested propelargonidin units presented here and elucidate additional structural information about these apparently unique proanthocyanidins. Herbivore studies using isolated *Cecropia* proanthocyanidins would provide information on their effects on herbivore growth and development that may reflect important chemically mediated ecological tradeoffs between the two species.
